# Perception and Attitude of Emergency Room Resident Physicians toward Middle East Respiratory Syndrome Outbreak

**DOI:** 10.1155/2017/6978256

**Published:** 2017-04-10

**Authors:** Mohammed Al Ghobain, Turki Aldrees, Abdullah Alenezi, Saleh Alqaryan, Dana Aldabeeb, Najed Alotaibi, Abdulrahman Aldhabib, Shaker Alghalibi, Sami Alharethy

**Affiliations:** ^1^Department of Medicine, College of Medicine, King Saud bin Abdulaziz University for Health Sciences, Riyadh, Saudi Arabia; ^2^Department of Otolaryngology-Head and Neck Surgery, College of Medicine, Prince Sattam Bin Abdulaziz University, Alkharj, Saudi Arabia; ^3^King Abdulaziz University Hospital, Riyadh, Saudi Arabia; ^4^College of Medicine, King Saud University, Riyadh, Saudi Arabia; ^5^Department of Emergency, King Khalid Hospital, Riyadh, Saudi Arabia; ^6^Department of Emergency, King Fahad Medical City, Riyadh, Saudi Arabia; ^7^Department of Otolaryngology-Head and Neck Surgery, College of Medicine, King Saud University, Riyadh, Saudi Arabia

## Abstract

*Introduction*. Middle East respiratory syndrome (MERS) outbreaks have had a considerable negative impact on health systems in Saudi Arabia. We aimed to study the psychological impact of a Middle East respiratory syndrome coronavirus (MERS-CoV) outbreak on emergency room resident physicians (ERRPs).* Methods*. We assessed the MERS-related psychological impact and concerns of ERRPs using a self-report questionnaire.* Results*. The majority (91%) of the ERRPs agreed that their work put them at risk of infection, but most (65%) did not agree that they should not be looking after patients infected with MERS. Despite that, 54% of ERRPs reported being afraid of contracting the infection from infected patients and only 4.2% of them were willing to change their current job. The majority of the ERRPs (85%) felt that their job would expose their families to risk of infection.* Conclusions*. Our study demonstrated the considerable psychological impact of MERS outbreaks on ERRPs. The ERRPs' concerns and the psychological impact of MERS outbreaks should be considered in greater detail by hospital policymakers.

## 1. Introduction

Middle East respiratory syndrome (MERS) is a deadly and contagious disease caused by a novel coronavirus (MERS-CoV) that has had an intensive impact on the healthcare system. The MERS-CoV can be transmitted in different ways, including direct contact with dromedaries, living with an infected person, or contact with infected persons inside healthcare facilities. The disease has a high mortality rate of up to 36% [1]. According to the World Health Organization (WHO), MERS is a threat to the entire world and thus needs prompt attention and action [1].

MERS was first identified in Saudi Arabia in 2012 [2]. Apart from Saudi Arabia, nearly 22 countries including Kuwait, Qatar, Indonesia, UK, UAE, South Korea, and USA have reported MERS cases. The WHO has reported 1,621 laboratory-confirmed cases of infection with MERS-CoV, and there have been nearly 584 deaths related to MERS-CoV since September 2012 [3]. Dromedary camels are the primary animal host for MERS-CoV [4, 5]. People with MERS have had a history of close contact with camels and drinking camel milk. However, human-to-human transmission has also been documented [6].

The majority of patients who become infected with the virus in the community seek their initial medical help from primary healthcare centers or from the emergency department in which emergency staff, including emergency physicians and nurses, are the first-line providers of care for suspected and unscheduled patients that require immediate attention. In addition, the emergency room resident physicians (ERRPs) are at risk of falling ill from exposure to MERS patients and the possibility of spreading the illness to their family members, which could affect their overall effectiveness and willingness to work during a MERS outbreak. Therefore, their anxiety and fears must be taken seriously and strategies should be incorporated to manage them. The MERS outbreak in Saudi Arabia was a very good opportunity to explore these concerns. To the best of our knowledge, no study has explored ERRPs' perceptions and attitude toward MERS or the psychological impact of MERS on ERRPs in hospitals. Thus, the present study aimed to assess the perceptions, attitude, and psychological impact of MERS on ERRPs.

## 2. Methods

A cross-sectional study was conducted using a self-administered questionnaire. The study was conducted in the emergency department after obtaining ethical approval from the King Saud University Research Center. All residents were informed regarding the purpose of the study and their verbal consent was given.

The study was conducted in four tertiary care hospitals in Riyadh, Saudi Arabia: King Khalid University Hospital, King Fahad Medical City Hospital, King Abdulaziz Medical City Hospital, and King Faisal Specialist. We included all ERRPs working in these four hospitals between November 2015 and December 2015 after a major outbreak of coronavirus (MERS-CoV) in Saudi Arabia.

Data were collected using a self-administered questionnaire. The questionnaires were distributed and collected during the regular weekly residents' education activities. The questionnaire contained a brief description of the study's objectives and statements assuring participants of their anonymity and right to stop participating of their own volition. The questionnaire was adapted and modified, based on a review of the literature, from one used in a previous study by Wong et al. [7]. The questionnaire comprised two parts. The first part contained questions about participants' background information, including age, marital status, training level, and knowledge of MERS. The second part comprised four main sections that included an assessment of (1) work-related concerns, (2) non-work-related concerns, (3) the perceived impact of MERS on ERRPs, and (4) their preparedness for a MERS outbreak.

All of the descriptive data were summarized as percentages and frequencies. Categorical variables were compared using the chi-square test. All statistical analyses were performed using IBM SPSS Statistics 21 (IBM Inc., Armonk, NY).

## 3. Results

The total sample size of this study was 94 ERRPs (out of 137 ERRPs; response rate 69%). The ERRPs had a mean age of 27 years, 65% were male, and 37% were married. Fifty-six percent of the ERRPs rated their knowledge about MERS-CoV as inadequate while 63% had had direct contact with at least one patient infected with MERS-CoV (Table 1).

The majority of the ERRPs (91%) agreed that their work put them at risk of infection, but most (65%) did not agree that they should not be looking after patients infected with MERS. Despite that, 54% of ERRPs were afraid of contracting the infection from infected patients and only 4.2% of them were willing to change their current job. The majority felt that their job would expose their families (85%), parents (83%), and close friends (77%) to risk of infection (Table 2).

Table 3 summarizes the perceived impact of MERS-CoV and readiness for an outbreak. Of the ERRPs, 48% would be afraid of telling their families about the risk of exposure, 42% agreed that there would be inadequate staff in the workplace to handle the increased demand, and 42% would feel more stressed at work. Furthermore, 57% of ERRPs had received infection control training and 55% reported that their hospitals have enough infection control staff to deal with such an outbreak.

There were no significant differences in level of concern by rotation except that first year training levels residents were more likely to be afraid of being infected than other training levels (*p* < 0.003). There were also no significant differences in terms of knowledge of MERS but respondents who had previously provided care for MERS patients were more likely to agree that the job put them at risk and were more afraid of being infected (*p* = 0.016 and *p* = 0.040, respectively) (data not shown in tables).

## 4. Discussion

Our study highlights the perceptions, attitude, and psychological impact of MERS outbreaks among ERRPs in Saudi Arabia. As MERS was a new outbreak that began in Saudi Arabia, to our knowledge, this was the first study to address the impact of a MERS outbreak among ERRPs. The findings of our study showed that the majority of ERRPs believed that their work put them at risk of infection and they were afraid of contracting MERS. The majority of the ERRPs felt that their job would expose their families, parents, and close friends to risk of infection. A small pilot study conducted in a single center in Jeddah, Saudi Arabia, in which the majority of respondents were nurses from the Philippines, did address the emotions and coping strategies of the healthcare workers who faced a MERS-CoV outbreak. It was found that the main sentiments centered upon fear for personal safety and the well-being of colleagues and family. Their fear was alleviated by positive attitudes in the workplace, clinical improvement of infected colleagues, and stoppage of disease transmission among healthcare workers after adopting strict protective measures. The most important emotion that drove them to continue working was their ethical and professional obligation toward their profession [8].

Our findings are similar to those of other non-MERS viral outbreaks reported from different parts of the world. For instance, during the severe acute respiratory syndrome outbreak (SARS) in 2003, approximately two-thirds of healthcare workers in Toronto experienced concern for their own or their family's health [9] while 68% of healthcare workers in Hong Kong reported a high level of stress and 57% were found to have experienced psychological distress [10]. A study of the psychological impact of the 2003 outbreak of SARS on hospital employees in Beijing, China, found that about 10% of the respondents had experienced high levels of posttraumatic stress symptoms [11].

An interesting finding of our study was that most ERRPs did not agree that they should not be looking after patients infected with MERS. We think that these finding are reflections of the high standards of ethical values of ERRPs and their professional obligation toward their jobs and patients.

In contrast, a study conducted, in Australia, anticipated a high rate of absenteeism among hospital staff, reaching up to 36% in response to influenza pandemic hypothesized survey scenario [12].

More than half of ERRPs had attended infection control training, and they reported that their hospitals had enough infection control staff to deal with such an outbreak and their hospitals have a clear plan to handle a MERS outbreak. This is a positive aspect and a positive learning experience from a MERS outbreak as it reflects good preparation and a high level of awareness of dealing with such an outbreak. How hospitals plan to handle a MERS outbreak was included but not limited to cancellation of outpatient clinics, visitor restrictions, mandatory wearing of protective measures (e.g., masks), limited hospital entrance, mandatory screening of suspect infected visitors, and public awareness and education.

The limitations of our study include it having a cross-sectional design, possible reporting bias, and being conducted in a single city in Saudi Arabia with a small sample size limited to a single profession (i.e., ERRPs). However, the study was conducted in four major hospitals in Saudi Arabia that all treated and handled patients with MERS infection, which justifies the generalization of our findings. We limited our study to ERRPs, as we believe that they are the healthcare workers at the highest risk of exposure because they deal with suspected cases before the diagnosis is established. The sample size was representative and reflects the real number of ERRPs working in these hospitals during the outbreak.

## 5. Conclusions

Our study demonstrated the considerable psychological impact of MERS outbreaks on ERRPs. The ERRPs' concerns and the psychological impact of MERS outbreaks should be considered in greater detail by hospital policymakers.

## Figures and Tables

**Table 1:** The participants' demographic characteristics.

	*N* (%)
Age (years)	27 (26–29)
Sex	
Male	61 (65)
Female	33 (35)
Marital status	
Unmarried	59 (63)
Married	35 (37)
Training level	
R1	44 (46.8)
R2	14 (14.9)
R3	19 (20.2)
R4	17 (18.1)
Knowledge of MERS-CoV	
Inadequate	53 (56)
Adequate	41 (44)
Provided care to a MERS patient	
No	35 (37)
Yes	59 (63)

MERS: Middle East respiratory syndrome; MERS-CoV: Middle East respiratory syndrome coronavirus. R1: first year resident; R2: second year resident; R3: third year residents; R4: fourth year resident.

**Table 2:** Work- and non-work-related concerns of emergency room resident physicians regarding Middle East respiratory syndrome.

Concerns	Disagree *N* (%)	Neutral *N* (%)	Agree *N* (%)
*Work-related concerns*			
My job would put me at great risk of exposure (*n* = 94)	2 (2.1)	6 (6.3)	86 (91.5)
I am afraid of being infected with MERS (*n* = 94)	19 (21)	24 (25)	51 (54)
I should not be looking after MERS patients (*n* = 94)	61 (65)	21 (22)	12 (13)
I might change my job because of the risk (*n* = 94)	82 (87)	8 (8.5)	4 (4.2)
*Non-work-related concerns*			
Family (*n* = 44)	5 (10)	2 (5)	37 (85)
Parents (*n* = 92)	9 (10)	7 (7)	76 (83)
Close friends (*n* = 91)	9 (10)	12 (13)	70 (77)
Work colleagues (*n* = 91)	9 (10)	12 (14)	70 (76)
People close to me would be worried about my health (*n* = 90)	5 (5)	13 (15)	72 (80)

*Note.* MERS: Middle East respiratory syndrome.

**Table 3:** Percentages of responses to perceived impact on personal life, workload, and preparedness for a Middle East respiratory syndrome coronavirus outbreak by emergency room resident physicians.

Perceived impact	Disagree (%)	Neutral (%)	Agree (%)
I would be afraid of telling my family about the risk I am exposed to (*n* = 94)	24	28	48
People would avoid me because of my job (*n* = 93)	56	20	24
There would be inadequate staff in my workplace to handle the increased demand (*n* = 94)	25	33	42
I would feel more stressed at work (*n* = 94)	38	20	42
I would have an increase in workload (*n* = 92)	32	27	41
I have attended infection control training sessions (*n* = 93)	34	9	57
In our hospital, we have enough infection control staff (*n* = 93)	15	30	55
Our hospital has a clear plan to handle a MERS outbreak (*n* = 93)	11	25	64
Received adequate personal protective equipment training (*n* = 93)	15	17	68
I am personally prepared for MERS (*n* = 93)	19	26	55

MERS: Middle East respiratory syndrome.
